# The Atypical Stimulant and Nootropic Modafinil Interacts with the Dopamine Transporter in a Different Manner than Classical Cocaine-Like Inhibitors

**DOI:** 10.1371/journal.pone.0025790

**Published:** 2011-10-17

**Authors:** Kyle C. Schmitt, Maarten E. A. Reith

**Affiliations:** 1 Department of Pharmacology, New York University School of Medicine, New York, New York, United States of America; 2 Department of Psychiatry, New York University School of Medicine, New York, New York, United States of America; Chiba University Center for Forensic Mental Health, Japan

## Abstract

Modafinil is a mild psychostimulant with pro-cognitive and antidepressant effects. Unlike many conventional stimulants, modafinil has little appreciable potential for abuse, making it a promising therapeutic agent for cocaine addiction. The chief molecular target of modafinil is the dopamine transporter (DAT); however, the mechanistic details underlying modafinil's unique effects remain unknown. Recent studies suggest that the conformational effects of a given DAT ligand influence the magnitude of the ligand's reinforcing properties. For example, the atypical DAT inhibitors benztropine and GBR12909 do not share cocaine's notorious addictive liability, despite having greater binding affinity. Here, we show that the binding mechanism of modafinil is different than cocaine and similar to other atypical inhibitors. We previously established two mutations (W84L and D313N) that increase the likelihood that the DAT will adopt an outward-facing conformational state—these mutations increase the affinity of cocaine-like inhibitors considerably, but have little or opposite effect on atypical inhibitor binding. Thus, a compound's WT/mutant affinity ratio can indicate whether the compound preferentially interacts with a more outward- or inward-facing conformational state. Modafinil displayed affinity ratios similar to those of benztropine, GBR12909 and bupropion (which lack cocaine-like effects in humans), but far different than those of cocaine, β-CFT or methylphenidate. Whereas treatment with zinc (known to stabilize an outward-facing transporter state) increased the affinity of cocaine and methylphenidate two-fold, it had little or no effect on the binding of modafinil, benztropine, bupropion or GBR12909. Additionally, computational modeling of inhibitor binding indicated that while β-CFT and methylphenidate stabilize an “open-to-out” conformation, binding of either modafinil or bupropion gives rise to a more closed conformation. Our findings highlight a mechanistic difference between modafinil and cocaine-like stimulants and further demonstrate that the conformational effects of a given DAT inhibitor influence its phenomenological effects.

## Introduction

Modafinil (2-(benzhydrylsulfinyl)acetamide) is a mild psychostimulant-like agent that increases wakefulness, improves attention and enhances performance in a variety of cognitive tasks [1]–[3]. Modafinil has been shown to exert antidepressive effects [4] and like other stimulants is an effective adjuvant for those experiencing only marginal improvement with serotonergic compounds [5], [6]. Classical psychostimulants, such as dextroamphetamine and methylphenidate exhibit dose-dependent biphasic effects on cognition—enhancing performance, learning and memory consolidation at moderate doses, but impairing cognitive function when used at high doses [7]–[9]. From a phenomenological perspective, modafinil has nootropic (pro-cognitive) effects similar to those of low-dose classical psychostimulants. However, compared to typical stimulants, modafinil possess a far more subtle and benign pharmacological profile [10]. Modafinil appears to lack many of the undesirable side effects of other stimulants, most notably: cardiovascular strain, sympathomimetic peripheral stimulation and significant addictive liability [11]. As such, modafinil has shown considerable promise as a therapeutic in the treatment of addiction to cocaine, one of the most frequently-used recreational drugs and likely the most addictive, based upon the percentage of both initial and regular users that transition into severe addicts [12], [13]. Modafinil attenuates craving for cocaine during drug withdrawal and has also been shown to decrease self-administration of smoked cocaine base (crack) in habitual crack users [14], [15]. Importantly, a recent study of modafinil self-administration in human cocaine addicts demonstrated that modafinil was not administered more frequently than placebo, nor did it occasion cocaine-like subjective effects [16].

The pharmacodynamic mechanism of modafinil is rather poorly understood and a wide-ranging variety of neurochemical systems have been previously implicated in its activity (for review, see *e.g.* [17]). One of the most prominent unresolved questions regarding modafinil's mechanism of action is: why does it lack the notable addictive potential of classical stimulants, such as cocaine? An understanding of why modafinil has a far lower abuse liability than prototypical stimulants may facilitate the design of novel and improved stimulant therapeutics for ADHD, cognitive enhancement, depression and cocaine addiction. In order to address this question, however, one must first possess insight into the protein target(s) of modafinil in the brain. Zolkowska *et al.* (2009) recently performed a “receptorome” screen, examining the interaction of modafinil with a large array of different neuronal receptor and transporter proteins in vitro [18]. Of the included receptor proteins, the neuronal dopamine transporter (DAT) was the sole target at which modafinil displayed relevant binding (that is, the only protein for which it possessed a *K*_i_ value lower than the threshold of 10 µM). However, the addictive stimulants cocaine and methylphenidate also principally target the DAT. What makes modafinil different? One enigmatic aspect of DAT pharmacology is the disparate reinforcing efficacy of various transporter ligands. A particular DAT-inhibiting molecule may have dramatic, mild or even a complete lack of behaviorally rewarding effects, regardless of absolute binding affinity [19], [20]. In this sense, the DAT appears to behave somewhat like a classically defined receptor, in that interaction with chemically distinctive ligands can elicit different behavioral effects in vivo. Recently, different chemical classes of ligands have been shown to stabilize the transporter protein in distinct conformational states upon binding; moreover, interaction with a specific conformation has been posited to affect the “addictiveness” of a given ligand [21]. It is important to note that rate of onset has also been shown to affect the addictiveness of DAT ligands—compounds with a rapid onset of action tend to exhibit greater reinforcing efficacy than those with a slower onset rate [22]–[25]. Compared to cocaine, modafinil has a slower onset of action [26]; hence, it is possible that this characteristic also contributes to its low addictive liability.

**Figure 1:**
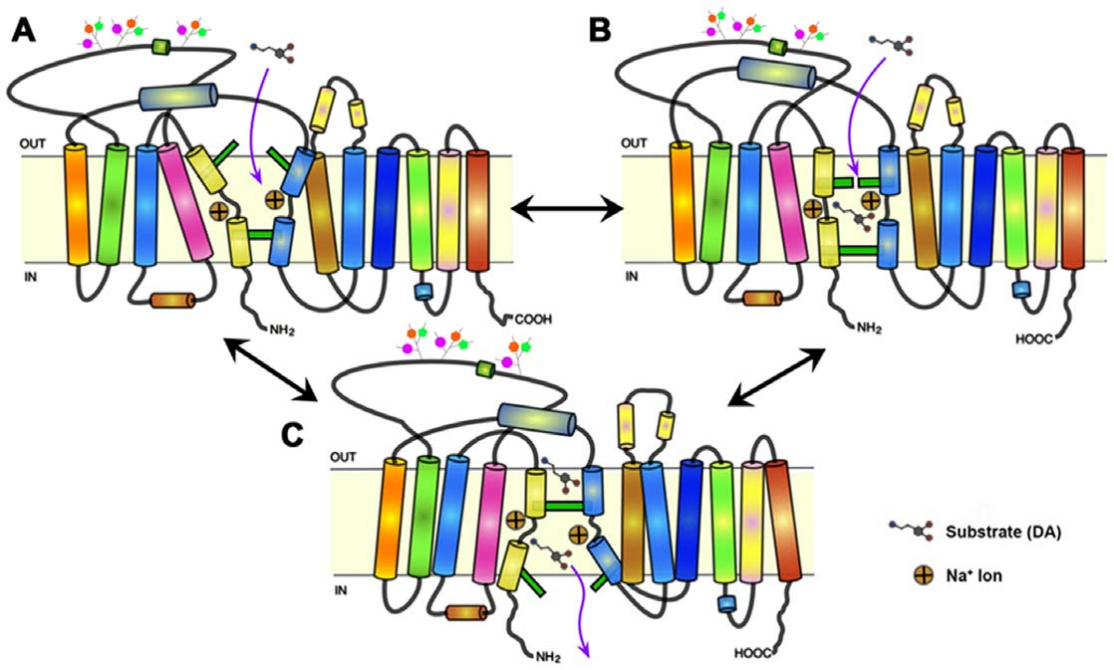
Cartoon representation of the DAT alternating access conformational cycle. (**A**) A fully outward-facing conformation with an open extracellular gating network (open-to-out) is established by binding of Na^+^ at the S1 site and is therefore the predominant state in the presence of high extracellular Na^+^ levels and absence of substrate. (**B**) Following Na^+^ binding, substrate interaction with S1 site residues triggers closure of the extracellular gate, establishing an occluded (closed-to-out) intermediate conformation. (**C**) Putative interaction of a second molecule of substrate with the vestibular S2 site helps facilitate opening of the intracellular gating network, giving rise to a fully inward-facing (open-to-in) conformation capable of releasing S1-bound substrate and ions into the cytoplasm.

The specific molecular mechanism underlying the DAT's substrate translocation cycle is not known. However, high-resolution crystallographic structures of a related transporter protein—a leucine transporter from the bacterium *Aquifex aeolicus* (LeuT)—bound to a variety of substrate-like and inhibitor-like ligands [27]–[29] provided a groundbreaking template for *in silico* molecular modeling of DAT ligand-binding dynamics [30], [31]. LeuT is a prokaryotic member of the neurotransmitter/sodium symporter (NSS) family of proteins, which also includes the eukaryotic transporters for serotonin, noradrenaline and dopamine (SERT, NET and DAT, respectively). The crystal structures, combined with a plethora of additional investigations of LeuT binding kinetics [32], [33] and single-molecule dynamics [34], [35] suggest an alternating access translocation cycle with at least three dominant low-energy conformational states (depicted in Fig. 1). The substrate interaction pocket at the center of the 12 transmembrane domain (TM) transporter protein (referred to as the ‘S1’ or primary substrate site) can be occluded from solution by both intra- and extracellular gating networks. These gates are formed by a small number of critical residue side-chains (highly-conserved throughout the NSS family), via networks of ionic, π-cation and hydrogen-bonding interactions [36]. Disruption and reformation of these interaction networks—mediated by the binding of ions and substrate or other ligands [34]—likely underlies the alternating access mechanism, allowing transition between terminal “open-to-out” (outward-facing) and “open-to-in” (inward-facing) conformations, with a dually occluded intermediate. Further studies with LeuT have revealed the presence an additional substrate-binding domain (dubbed the ‘S2’ site) located in the extracellular vestibule of the transporter, 11–13 Å above the central S1 site. This vestibular site appears to bind a variety of different ligands, including a second molecule of the substrate leucine [32], alkylglucoside detergents [37] and a variety of antidepressant compounds, both tricyclics [28], [38] and SSRIs like fluoxetine and sertraline [39]. Interestingly, whereas tricyclics and other inhibitors that bind at the S2 site stabilize LeuT in an occluded state, binding of the competitive inhibitor tryptophan (which binds at the S1 site, displacing leucine itself) stabilizes an open-to-out conformational state [29].

Mutagenesis and cysteine-accessibility studies suggest that cocaine and structural analogues preferentially stabilize the DAT in the open-to-out conformation [40], [41]. In contrast, atypical inhibitors—compounds that potently inhibit the DAT, yet do not share cocaine's abuse potential (such as benztropine, GBR12909 and bupropion)—stabilize a “closed-to-out” conformation; that is, either an occluded or inward-facing state [21], [42]. Here, we present evidence that modafinil displays atypical-like binding characteristics—stabilizing the DAT in a different conformation than cocaine-like compounds. We have previously characterized two DAT mutations (W84L and D313N) that disrupt the transition between outward- and inward-facing states, increasing the likelihood that the transporter will adopt an outward-facing conformation [43]. These mutations considerably increase the affinity of cocaine-like inhibitors as measured by inhibition of [^3^H]CFT binding, but have negligible or opposing effects on the affinity of atypical inhibitors [42], [44]. Thus, a given DAT ligand's affinity ratio at mutant versus WT transporters can offer insight into whether the ligand preferentially interacts with the outward- or the inward-facing conformational state. We employed these mutants, as well as conformation-biasing ionic conditions [45], to investigate the binding mechanism of modafinil at the DAT. Additionally, we performed *in silico* induced-fit docking of the atypical inhibitors modafinil and bupropion and the cocaine-like inhibitors β-CFT and methylphenidate, in order to probe possible structural differences in DAT interaction between the two classes of compounds.

## Materials and Methods

### Generation of cell lines stably expressing WT and mutant DATs

In this work, we used Human Embryonic Kidney cells (HEK293) stably expressing WT human DAT, or the human DAT mutants W84L or D313N. HEK cells were obtained from ATCC (ATCC CRL 1573) as previously described; transfected cell lines were prepared by us for studies previously reported [43], [44]. Human DAT mutant plasmids were generated using site-directed mutagenesis as previously outlined [43]. Mutations were screened by PCR and restriction enzyme mapping. The cells were stably transfected with the various DAT plasmids using Lipofectamine (Invitrogen, Carlsbad, CA, USA) and were maintained with ∼250 µM geneticin (G418).

### [^3^H]CFT binding inhibition assays

For binding assays, suspensions of intact HEK-hDAT were prepared according to the method outlined previously [42], [44]. Cell slurry was incubated for 1 hr at 21°C and centrifuged; the supernatant was discarded and the subsequent pellet was washed and gently resuspended in 6 mL KRH buffer solution in preparation for assay. Modified Krebs/Ringer/HEPES (KRH) buffer containing 1 mM ascorbic acid and 0.1 mM tropolone was used. In the ‘sodium free’ binding conditions, buffer NaCl was isotonically replaced with *N*-methyl-D-glucamine chloride (NMDG-Cl). For zinc-modulated binding conditions, 10 µM Zn^2+^ was added to the assay buffer before the addition of the unlabeled test ligand and [^3^H]CFT. Assays were conducted in 96-well plates at 21°C, with all determinations performed in triplicate wells. Binding reactions were initiated by addition of 50 µL cell suspension to buffer containing radioligand and varying concentrations of test ligand, for a final per-well reaction volume of 200 µL. Cells were incubated with 2–4 nM [^3^H]CFT (85.9 Ci/mmol) and test compounds for 15 min at 21°C. Nonspecific binding was determined using 1 µM non-radiolabeled β-CFT. Binding was terminated by vacuum filtration onto a filtermat (Wallac A) and washing with 0.9% ice-cold saline using a Tomtec automatic 96-pin cell harvester (Tomtec, Orange, CT, USA). Tritium accumulation was quantified using a Microbeta 1405 liquid scintillation counter (Perkin-Elmer, Boston, MA, USA).

### Data analysis and statistics

Kinetic parameters, such as the equilibrium dissociation constant of radioligand binding (*K*_D_), were determined by respective competition analysis with non-radiolabeled β-CFT, using Kell RADLIG (Biosoft, Cambridge, UK). For each tested DAT ligand, the IC_50_ for inhibition of [^3^H]CFT binding was calculated with Origin 7.5. IC_50_ values for the DAT ligands were converted into relative inhibition constants (*K*_i_) using the Cheng-Prusoff equation [46].

### Homology modeling and flexible docking

The DAT protein homology model was generated in a manner similar to the procedure detailed in Schmitt *et al.* (2010) [47]. The crystal structure of LeuT bound to the ligands leucine and the tricyclic antidepressant (TCA) desipramine ([28]; PDB Index 2QJU) was used as the structural template, employing the NSS-family protein amino acid sequence alignment proposed by Beuming *et al.* (2006) [48]. Since the sequence of LeuT is shorter than that of the DAT, parts of the intracellular termini were excluded from the model (N-terminal residues M1-V55 and residues K589-V620 on the C-terminus). In addition, all water and β-octylglucoside molecules and the ligands present in the template LeuT crystal were not included in the DAT model. The sodium ions were initially placed in the DAT model based upon their location in LeuT, but were allowed to move freely during energy minimization, docking and optimization rounds. The DAT chloride ion was initially placed at the position corresponding to E290 in the LeuT structure (in the DAT, this residue is S357—the negative charge provided by glutamate renders LeuT Cl^−^-insensitive) [49], [50].

Homology modeling was performed using the MODELLER algorithm and the resultant lowest energy structure was imported into the Molecular Operating Environment (MOE) program suite (Version 2009.10; Chemical Computing Group, Montreal, CA). The Protonate3D function in MOE was used to calculate residue protonation states and assign hydrogen atom coordinates; partial charges were assigned according to the AMBER99 forcefield. In order to refine residue stereochemistry and relieve any steric clashes in the protein prior to ligand docking, the DAT model was subjected to several rounds of energy minimization, employing the AMBER99 forcefield and the generalized Born (GB/VI) implicit solvation model [51]. During the first round, protein backbone atoms were dynamic and the model was minimized until hitting a convergence gradient of 0.05 kcal mol^−1^ Å^−1^. Subsequent minimization rounds focused on optimizing side chain geometry of particular residues, hence backbone atoms were tethered and a more stringent convergence value (0.001 kcal mol^−1^ Å^−1^) was employed. Analysis of the final DAT model with PROCHECK [52] indicated that 98.9% of the residues fell within either the ‘most favored’ or ‘additionally allowed’ Ramachandran plot region (86.8% most favored); only four residues (0.9%) fell within the ‘generously allowed’ region and only one residue (Q373; 0.2%) was deemed to be in the ‘disallowed’ region (for further discussion of model stereochemical quality, see [47]).

Ligand binding sites in the DAT model were identified with the Site Finder tool implemented in MOE—after manual elimination of sites lying directly on the exterior, cytoplasmic or extracellular faces of the protein, two binding pockets (approximately overlapping with the S1 and S2 sites of LeuT) were identified. Dummy atoms were placed at the centroids of alpha spheres defining these two sites to assist in ligand docking. For docking, ligand structures were imported into MOE, protonated, assigned partial charges and energy minimized (<0.001 kcal mol^−1^ Å^−1^) using the MMFF94x forcefield with GB/VI implicit solvation. In the preliminary docking process, ligand bond length and DAT protein atoms are held constant and various ligand orientations and conformational rotomers are systematically positioned in the active site such that no steric clashes between ligand and residue side-chains occur. The top 50 non-duplicate docked poses (London dG scoring method) were output to a MOE database and manually sorted into two population clusters, representative of binding at either the central S1 site (below the R85-D476 gating interaction) or the vestibular S2 site (above the R85-D476 gate). Examples of S1-localized highly-populated “metapose” clusters are shown in the Supporting Information (Fig. S1). An energetically favorable (top-scoring) pose from each population was chosen as a representative for ligand-adaptive geometric optimization; however, poses that did not display any strong molecular interactions (*e.g.* hydrogen bonds, cation-π and aromatic π-stacking interactions) with specific residues within their binding pocket were not considered.

Representative poses were then refined by further minimization of the protein/ligand. In refinement rounds, protein backbone atoms were weakly tethered (1 kcal mol^−1^ Å^−1^ force constant) and the side-chain and ligand atoms completely unconstrained to allow for flexible “ligand adaptive” docking—for the last minimization round, the backbone tethering constant was increased to 10 kcal mol^−1^ Å^−1^ and the convergence gradient was set at 0.01 kcal mol^−1^ Å^−1^. Final ray-traced models depicted in figures were rendered with PyMOL 1.4 (Schrödinger LLC, New York, NY, USA). All MOE simulations were performed on a standard quad-core ×64 computer running Windows 7.

## Results

### Binding and mutant affinity-shift profile of modafinil and DAT inhibitors

**Figure 2:**
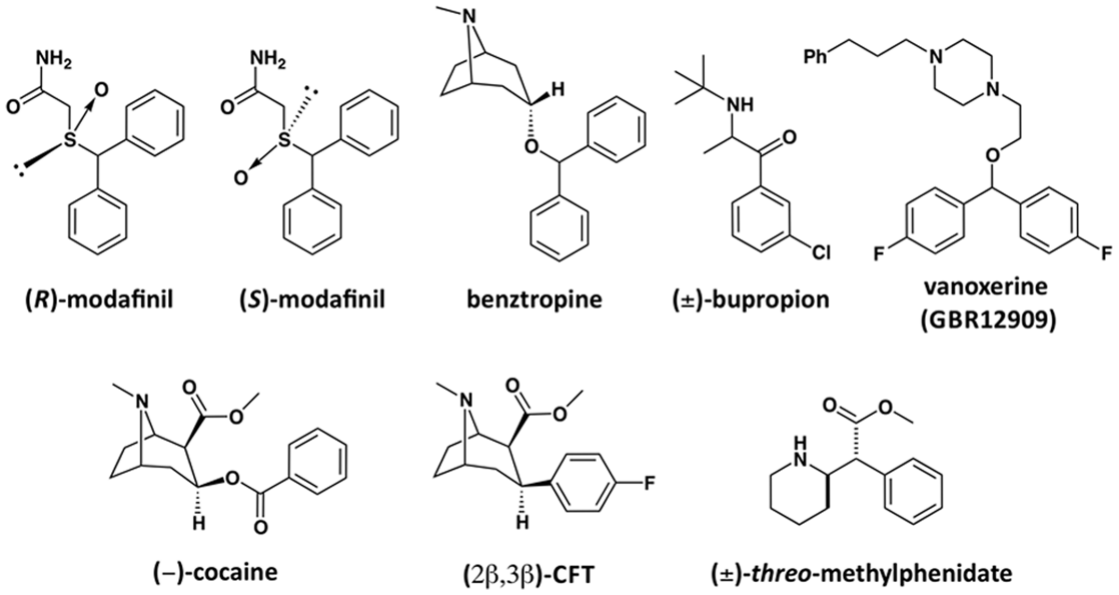
Chemical structures of modafinil and other tested DAT inhibitor ligands. Atypical inhibitors (top row) exhibited preferential interaction with a more inward-facing transporter conformation, whereas cocaine-like inhibitors (bottom row) preferentially bound to the outward-facing DAT conformation. While modafinil has a chiral sulfoxide moiety, the enantiomers possess little difference in pharmacodynamic activity (hence, only the racemate was tested).

Modafinil and other compounds—representing different chemical classes of DAT ligands (Fig. 2)—were assayed for their ability to inhibit [^3^H]CFT binding to WT or mutant DATs expressed in whole HEK293 cells. The binding affinities (*K*_i_ values) of the tested compounds and the observed WT/mutant affinity ratios are listed in Table 1. Modafinil's binding affinity at WT transporters was relatively low (*K*_i_ = 2.1 µM); compared to the other reference ligands, modafinil was anywhere from 6- to 100-fold weaker (Table 1). The micromolar level affinity is consistent with prior literature reports of modafinil radioligand binding at the DAT and likely underlies the comparatively high effective dose of modafinil (200–600 mg) in humans [18], [53]. At the W84L mutant, modafinil showed a significant decrease in affinity (an increase in *K*_i_ value to 3.8 µM; *p*<0.05) compared with the WT transporter, resulting in a WT/W84L *K*_i_ ratio of 0.56 (Table 1). This mutant affinity-shift was strikingly similar to that observed with the atypical ligands benztropine, GBR12909 and bupropion (for each of these ligands, the WT/W84L *K*_i_ ratio was approximately 0.5). In contrast, the classical DAT inhibitors cocaine, β-CFT and methylphenidate all showed significantly increased binding affinity (decreased *K*_i_ value) at the W84L mutant: the tropane compounds both gave 3.5-fold improvements, whereas methylphenidate displayed a more modest 2-fold gain. At the D313N mutant, modafinil showed little change in affinity compared with WT (having a WT/D313N *K*_i_ ratio of 0.95), behaving similarly to bupropion and GBR12909—which gave WT/D313N *K*_i_ ratios of 0.90 and 1.05, respectively—but not to any of the cocaine-like ligands (Table 1).

**Table 1:** Potencies of modafinil and other DAT inhibitors, assessed by displacement of intact-cell [^3^H]CFT binding to WT or mutant hDAT.

Compound	Whole-Cell hDAT Binding *K*_i_ (nM)			*K*_i_ [WT]/*K*_i_ [Mutant] Ratio	
	WT	W84L	D313N	WT/W84L	WT/D313N
β-CFT	15.4±2.1	4.44±0.69*	6.14±0.29*	3.47	2.51
(−)-cocaine	163.6±1.20	46.7±4.52*	51.5±5.06*	3.50	3.18
(±)-methylphenidate	21.2±3.7	11.1±1.6*	11.4±0.38*	1.91	1.86
benztropine	75.3±7.4	189.5±6.82*	181.4±30.3*	0.40	0.42
(±)-bupropion	319.5±24.9	745.9±14.0*	353.9±16.7	0.43	0.90
GBR12909	53.2±19.7	108±10.7*	50.6±1.2	0.49	1.05
(±)-modafinil	2143±215	3816±266*	2255±229	0.56	0.95

Binding assays were performed using intact stably-transfected HEK293 cells; values are means ± SEM for 3–6 experiments, each performed in triplicate.

*Significant difference versus wild-type binding affinity (*p*<0.05; *t* test, two-tailed). Data for inhibitors other than modafinil included from [42] for reference.

### Effects of ionic conformational manipulation on modafinil and DAT inhibitor binding

Various endogenous ionic species are known affect the conformational equilibrium of the DAT and other NSS-family proteins. For example, recent biophysical studies with LeuT have demonstrated that binding of Na^+^ to the substrate-free (apo) form of the transporter induces a conformational shift toward the open-to-out state, increasing accessibility of the extracellular vestibule [34] and constricting residues near the intracellular gating network [35], [54]. The sodium gradient present under normal physiological conditions (high extracellular Na^+^ concentration and low intracellular Na^+^ concentration) therefore gives rise to a population of transporters that are predominantly outward-facing, primed to bind ligands approaching from the extracellular milieu [55]. In the absence of significant sodium levels, the transporter effectively shifts between outward and inward-facing conformations [35]. Hence, changing the ionic conditions by removing extracellular sodium (without grossly altering intracellular ionic components) would be expected to increase the preponderance of a “closed-to-out” state amongst the overall population of transporters. Applying this logic to the DAT, we performed intact-cell binding assays with buffer Na^+^ isotonically substituted for the inert and membrane-impermeant cation NMDG^+^ (yielding a functionally 0 mM concentration of extracellular Na^+^ without significantly affecting intracellular ionic conditions), a treatment previously demonstrated to increase the relative number of inward-facing DATs [56]. Replacement of buffer sodium resulted in a decrease of affinity (increase in *K*_i_ value) for all of the tested DAT inhibitors (compare *K*_i_ values of WT transporter in Table 1 to those of the Na^+^-Free condition listed in Table 2). However, amongst the inhibitors, modafinil and GBR12909 were least impacted by sodium depletion, displaying 1.4- and 1.8-fold increases in respective *K*_i_ values.

**Table 2:** [^3^H]CFT binding potency of modafinil and other DAT ligands in the absence of extracellular Na^+^ and the effect of Zn^2+^ on binding affinity.

Compound	Whole-Cell WT hDAT Binding *K*_i_ (nM)		Zn^2+^ Effect Ratio
	Na^+^-Free (0 mM) Buffer	Na^+^-Free+10 µM Zn^2+^	*K*_i_ [0 µM]/*K*_i_ [10 µM]
(−)-cocaine	415.3±41.1	229.2±27.7*	1.81
(±)-methylphenidate	252.9±24.1	98.92±8.94*	2.56
benztropine	231.1±17.0	209.9±18.1	1.10
(±)-bupropion	709.3±65.3	737.1±55.4	0.96
GBR12909	95.99±8.74	126.8±15.6	0.76
(±)-modafinil	2963±161	3470±261	0.85

Assays were performed in Na^+^-free conditions (buffer sodium was isotonically replaced with the impermeant cation NMDG^+^) in the presence and absence of 10 micromolar zinc; values are means ± SEM for 3–7 experiments, each performed in triplicate.

*Significant difference versus Na^+^-Free affinity value (*p*<0.05; *t* test, two-tailed).

Zinc is another important endogenous modulator of the DAT; *in vivo*, it forms organometallic coordinations with three residues at the top of the extracellular vestibule of the transporter (H193, H375 and E396). By loosely “grasping” these three residues on the external protein face, zinc likely impedes the transition between outward- and inward-facing conformations, biasing the equilibrium in favor of the outward-facing state [41]. Effects of exogenously-applied Zn^2+^ are observable experimentally at micromolar concentrations: Zn^2+^ increases the binding of β-CFT and cocaine [44], [45] and can partially overcome the effects of DAT mutations exerting an inward-facing conformational bias (the opposite of the W84L or D313N mutations), such as the Y335A [41], D345N [57] and W267L [58] mutants. We thus used Zn^2+^ to investigate the conformational preference of modafinil and the other DAT ligands. By increasing the population of outward-facing DATs and (at least partially) reversing the effect of extracellular Na^+^ depletion, zinc can highlight compounds that selectively bind to an outward-facing state. Under sodium-free buffer conditions, the addition of 10 µM Zn^2+^ significantly increased the binding affinity (decreased the *K*_i_ value) of cocaine and methylphenidate at WT transporters (Table 2). For inhibition of [^3^H]CFT binding by cold β-CFT, the presence of Zn^2+^ under sodium-free conditions increased the *B*_max_ value of labeled [^3^H]CFT by a factor of four, from 125±15.8 fmole/well to 502±78 fmole/well. The calculated absolute *K*_d_ values for the sodium-free and +10 µM Zn^2+^ conditions were not significantly different: 49.32±9.69 and 57.08±6.67, respectively. This zinc-mediated effect—alteration in the *B*_max_, but not the *K*_d_ kinetic parameter—has been demonstrated before in both Na^+^-free [58] and physionormal Na^+^ (130 mM) buffers [45], [59]. It is likely that the particular kinetic effects of micromolar Zn^2+^-levels depend on the specific assay protocol and nonlinear curve-fitting algorithm used. Addition of Zn^2+^, however, had little impact on the atypical DAT inhibitors overall (the ratio of *K*_i_ values obtained in the absence and presence of zinc was close to unity for each compound; Table 2). This finding suggests that unlike β-CFT, cocaine or methylphenidate, the interaction of modafinil (like GBR12909, benztropine and bupropion) with the DAT is far less dependent on the transporter assuming an open-to-out conformational state.

### Adaptive docking of modafinil and other inhibitors in an hDAT model

In an attempt to gain structural insight into the differential interactions of cocaine and modafinil with the DAT, we employed a homology model of the human DAT and docked (*R*)-modafinil, as well as (*S*)-bupropion, (*d*)-methylphenidate and β-CFT with a flexible ligand-adaptive docking procedure. Specific enantiomers of the various DAT inhibitors were used in order to simplify the docking protocol. The (*S*)-enantiomer of bupropion was selected based upon the stereoselective dopaminergic activity of its primary metabolite (*S*,*S*)-hydroxybupropion [60] and the comparatively greater isomeric potency of other (*S*)-cathinones [61], [62]. Dexmethylphenidate (the *threo*-(*R*,*R*)-isomer of methylphenidate) has been extensively shown to be wholly responsible for the DAT-mediated physiological effects of the racemate [63], [64] and was therefore selected for modeling. The stereochemistry of modafinil differs from other DAT ligands, as modafinil's stereocenter is not the typical asymmetric carbon atom, but a sulfinyl moiety (Fig. 2). Unlike other DAT ligands, which generally possess significant enantioselectivity, (*R*)- and (*S*)-modafinil show only mild differences in DAT affinity, with the (*R*)-enantiomer having marginally greater affinity [65]. In humans, racemic modafinil and (*R*)-modafinil are active at similar doses, but the (*R*)-isomer has a more stable pharmacokinetic profile [66] and was recently released to the market as an enantiopure drug (armodafinil); hence, it was selected as the more “active” isomer for docking. β-CFT was chosen over cocaine for its structural rigidity, as flexibility imparted by cocaine's benzoyloxy moiety prevented the docking procedure from converging upon particularly consistent pose clusters. The hDAT model was based upon the structure of LeuT co-crystallized with its substrate leucine, as well as the tricyclic antidepressant desipramine [28]. We previously employed this DAT model in docking of substrates and bivalent substrate-like inhibitors [47]. Two ligand-binding pockets identified in the hDAT model were used for docking—roughly corresponding with the S1 and S2 sites of LeuT—and each inhibitor was docked in both sites. A single candidate was selected from a cluster of top-scoring poses and used as the initial input for further energy minimization of the protein/ligand complex (see Fig. S1 for examples of pose clusters from which potential candidates were selected).

**Figure 3:**
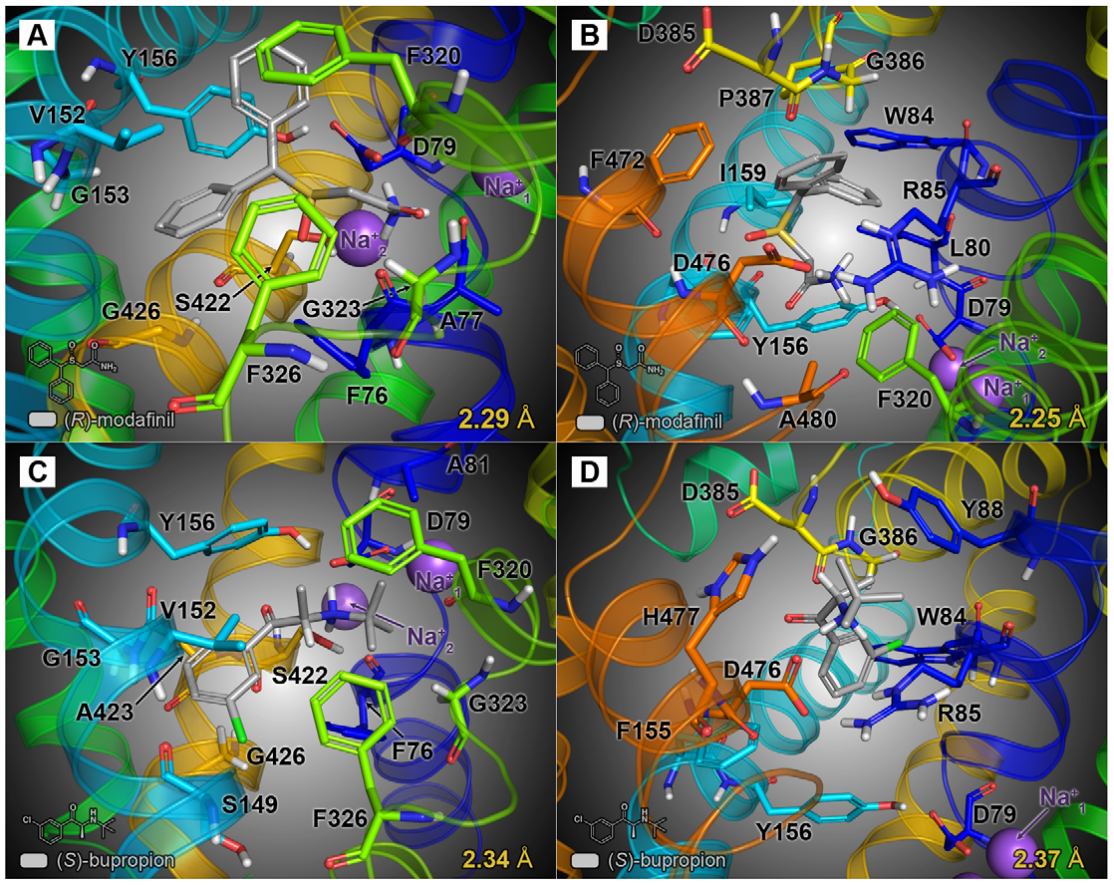
Final energy-minimized poses of atypical inhibitors docked at the DAT primary (S1) and vestibular (S2) substrate binding sites. Selected binding pocket residues are labeled and rendered as sticks; bound ligand molecules (also shown as sticks) are highlighted using gray-colored carbon atoms. The distance between the carboxylate oxygen atom of D79 and the ring hydroxyl moiety of Y156 is displayed in the lower right of each panel (in yellow). (**A, B**) (*R*)-modafinil docked at the S1 and S2 sites, respectively—at the S1 site (**A**), modafinil primarily interacts with D79 and adjacent TM1 residues, whereas at the S2 site (**B**), it mainly interacts with residues that form the extracellular gating network. (**C, D**) (*S*)-bupropion docked at both the S1 (**C**) and S2 sites (**D**). Note that for each of the DAT/inhibitor models, the bound inhibitor molecule does not disrupt the D79-Y156 hydrogen bond (*i.e.* the interatomic distance remains less than 3.5 Å following adaptive docking procedures).

**Figure 4:**
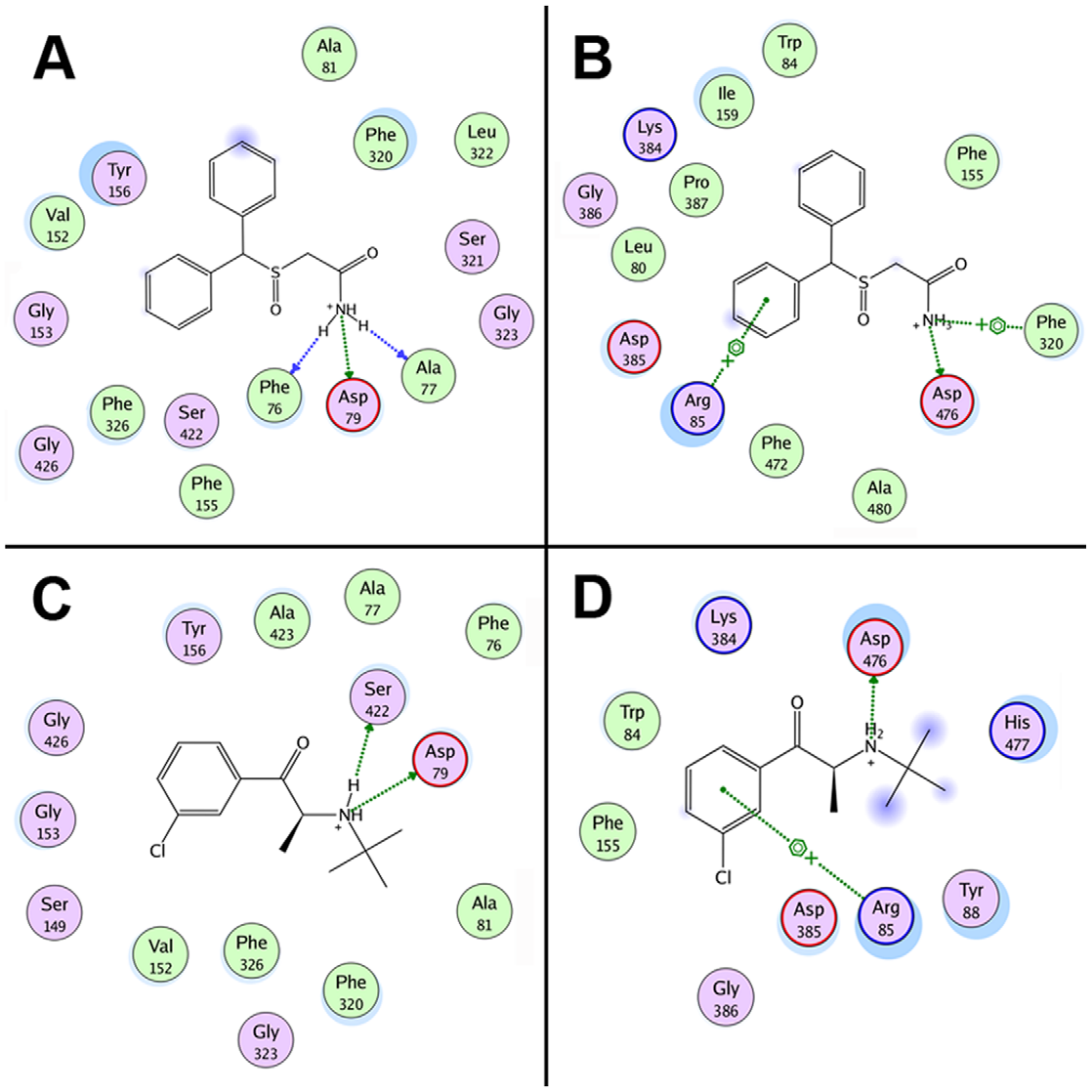
Molecular interaction diagrams of docked atypical inhibitors. For each panel, the interaction map depicts DAT residues located within 4.5 Å of the bound inhibitor molecule (hydrophobic residues are colored green and polar residues are purple). The most significant (non van der Waals) DAT/ligand interactions are indicated with dotted lines and a symbol depicting the chemistry of the interaction formed: side-chain hydrogen bond (green), main-chain hydrogen bond (blue), cation-π bond (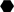
+) or aromatic π-stacking (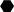 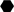). (**A, B**) Residue interaction maps for modafinil bound at the S1 (**A**) and S2 sites (**B**). (**C, D**) Interaction maps for bupropion bound at the S1 (**C**) and S2 sites (**D**), respectively. For both of the atypical inhibitors, binding at the S1 site (panels A and C) gives rise to few strong interactions with the DAT—only their protonated nitrogen atoms form hydrogen bonds—suggesting that recognition of these relatively modest inhibitors (*K*_i_>100 nM) is influenced more by molecular shape and steric bulk than by specific polar interactions.

Following docking at the S1 site, modafinil was oriented horizontally (parallel to the plane of the membrane), with the diphenyl ring system facing V152, G153 and Y156 of TM3 and the sulfinylacetamide chain surrounded by F76, A77, D79 of TM1 and F320, S321 and L322 of TM6 (Fig. 3A). In this pose, few strong molecular interactions between modafinil and the DAT were observed, save for hydrogen bonds formed between modafinil's terminal amide nitrogen and residues F76, A77 and D79 (Fig. 4A). At the S2 site, modafinil was positioned just above the extracellular vestibule gating residues R85, F320 and D476 (Fig. 3B); one phenyl ring formed a cation-π interaction with R85 and the protonated amide displayed a combination of hydrogen bonding with D476 and a cation-π interaction with the aromatic side chain of F320 (Fig. 4B). Bupropion docked at a slightly lower position in S1 (Fig. 3C), but like modafinil, the aromatic portion of the molecule was oriented parallel to V152 and enveloped by residues of TM3, whereas the amine nitrogen and bulky *tert*-butyl group were oriented towards D79, F320 and other adjacent residues of TMs 1 and 6 (Fig. 4C). In the S2 site, while bupropion was positioned marginally higher than modafinil in the extracellular vestibule (Fig. 3D), its strongest molecular interactions—a cation-π interaction with R85 and a hydrogen bond between the amine and D476—were similar (Fig. 4D).

**Figure 5:**
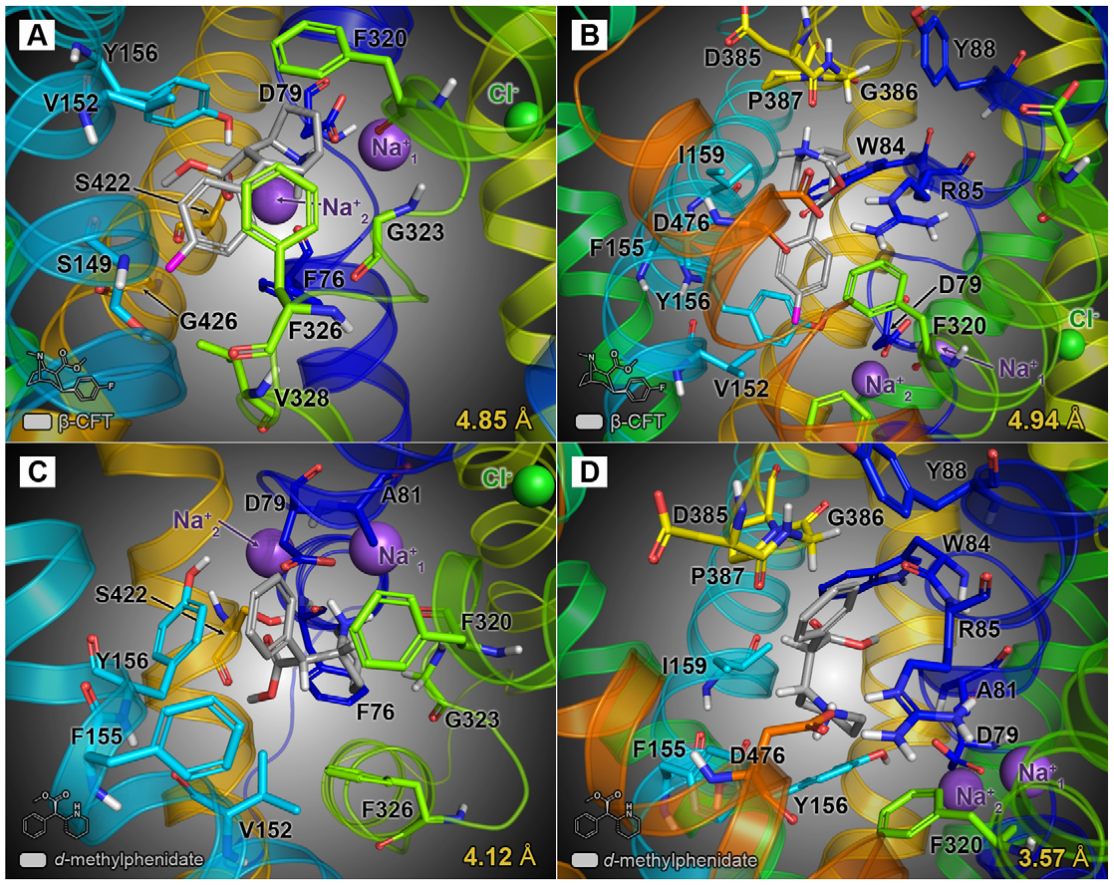
Final energy-minimized poses of cocaine-like inhibitors docked at the DAT S1 and S2 sites. Selected binding pocket residues are labeled and rendered as sticks; bound ligand molecules are highlighted using gray-colored carbon atoms. The distances between the oxygen atoms of D79 and Y156 are displayed in the lower right of each panel (in yellow). (**A, B**) β-CFT docked at the S1 (**A**) and S2 sites (**B**); binding of β-CFT at either site disrupts the hydrogen bond between and D79 and Y156 (interatomic distance >3.5 Å), indicating that it promotes an open-to-out conformational state. (**C, D**) Dexmethylphenidate docked at the respective S1 (**C**) and S2 sites (**D**)—similar to CFT, methylphenidate disrupts the D79-Y156 hydrogen bond upon binding at the S1 site (however, at the S2 site, the D79-Y156 interatomic distance is roughly ≈3.6 Å, hence the effect of methylphenidate on the integrity of the hydrogen bond is less conclusive).

**Figure 6:**
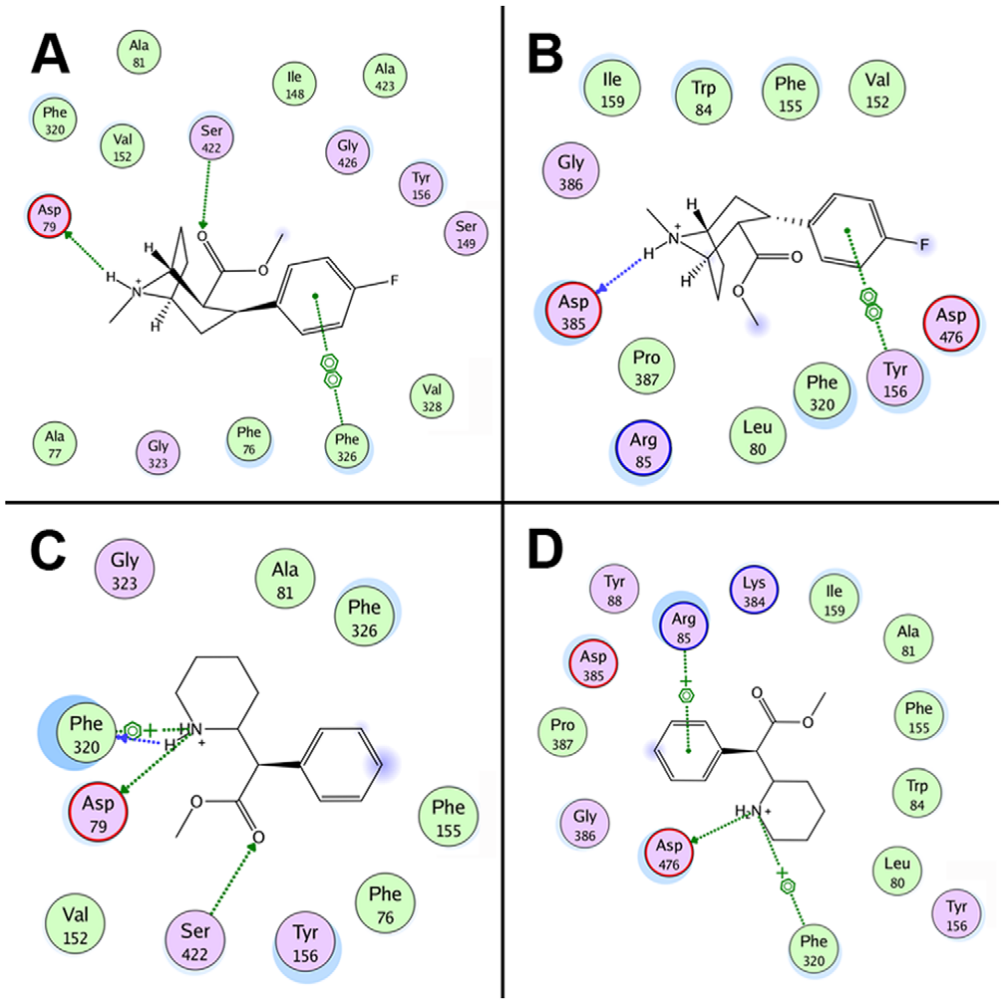
Molecular interaction diagrams of cocaine-like inhibitors docked at the S1 and S2 sites. For each panel, the interaction map depicts DAT residues located within 4.5 Å of the bound inhibitor. As described for Figure 4, the residues are colored based upon their chemical nature and the most significant DAT/inhibitor interactions are labeled with dotted lines and a symbol depicting the chemistry of the interaction formed. (**A, B**) Residue interaction maps for β-CFT bound at the S1 (**A**) and S2 sites (**B**). (**C, D**) Interaction maps for dexmethylphenidate bound at the S1 (**C**) and S2 sites (**D**), respectively. At the S2 site, the interaction pattern of methylphenidate is similar to that of modafinil (compare Figure 6D with Figure 4B).

The cocaine-like inhibitors β-CFT and *d*-methylphenidate also yielded highly populated pose clusters when docked in the S1 and S2 sites (a representative pose cluster for CFT docked at the S1 site is shown in Fig. S1B). At the S1 site, the tropane amine of CFT engaged in hydrogen bonding with D79, with the *N*-methyl group oriented downward towards F76 and neighboring residues in TMs 1 and 6 (Fig. 5A). The tropane ethylene bridge was directed upward toward the extracellular gate, likely blocking the aromatic side chain of F320 from establishing an interaction with the cationic nitrogen. In addition, the 3β-fluorophenyl ring of CFT participated in π-π stacking aromatic interaction with the side-chain of F326 and the 2β-carbomethoxy moiety formed a hydrogen bond with S422 of TM8 (Figs. 5A and 6A). Many of the interactions and binding pocket residues found for CFT were consonant with those reported in prior molecular simulations of phenyltropane binding at the S1 site (*e.g.* [31]). In the S2 site, CFT was oriented perpendicular to the plane of the membrane, with the charged tropane amine directed towards the top of the extracellular vestibule (Fig. 5B). Residues from extracellular loop 4 (D385, G386 and P387) helped to shield CFT from the extracellular space, with the backbone of D385 forming a hydrogen bond with the tropane nitrogen (Fig. 6B). The 2β-carbomethyoxy moiety was situated directly adjacent to the side-chains of R85, F155 and D476, but did not disrupt the interaction between R85 and D476. In contrast to the other DAT inhibitors docked in the S2 site, the aromatic portion of CFT dipped below the R85-D476 extracellular gate (Fig. 5B), enabling a π-π stacking interaction between the S1-localized residue Y156 and the 3β-fluorophenyl substituent (Fig. 6B). This binding orientation is relatively consistent with other computational studies modeling cocaine and phenyltropane binding in the extracellular vestibule (S2 site) of the dopamine and noradrenaline transporters in the presence of respective substrates bound at S1 [67], [68].

Despite adopting a slightly different orientation, we found that *d*-methylphenidate shared many of the same interactions and binding pocket residues with β-CFT when docked at the S1 site (Fig. 5C). In particular, the methyl ester moiety of methylphenidate engaged in hydrogen bonding with the side-chain of S422 and the cationic amine formed a bond with D79 (Fig. 6C). The greatest difference in the binding models of the two inhibitors involved F320: for methylphenidate, the charged piperidine amine group formed both a cation-π interaction with the aromatic side-chain of F320 and a hydrogen bond with the backbone. However, at the S2 site, methylphenidate exhibited an interaction pattern and binding orientation more akin to that of modafinil—forming a cation-π interaction between the ligand aromatic ring and R85, with the protonated ligand amine anchored by a combination of hydrogen bonding with D476 and a cation-π interaction with the aromatic side chain of F320 (Figs. 5D and 6D).

Our *in silico* modeling data are also consistent with the idea that modafinil interacts with the DAT in a different manner than cocaine-like inhibitors. In a recent study combining molecular simulation and site-directed mutagenesis, Beuming *et al.* (2008) showed that the presence or absence of a hydrogen bond between D79 and Y156 in a given DAT/ligand complex can provide an indication of the conformational bias engendered by the ligand [31]. The highly conserved TM3 tyrosine residue Y156 interacts with the substrate dopamine as it binds at the S1 site and also participates in the vestibular gating network—consisting of R85, F320 and D476—that partitions the S1 and S2 sites [69], [70]. When dopamine is bound at the S1 site, a hydrogen bond formed between the side chain oxygen atoms of D79 and the hydroxyl moiety of Y156 helps to close the extracellular gate, protecting the S1-bound substrate from infiltration by water from the extracellular space [31]. Hence, the presence of a D79-Y156 hydrogen bond is associated with a “closed-to-out” transporter state. In their molecular dynamics simulations, Beuming *et al.* (2008) showed that an interatomic distance of less than 3.5 Å (indicative of an intact hydrogen bond) was maintained between the oxygen atoms of D79 and Y156 during binding of DAT substrates (dopamine, amphetamine and MDMA) in the S1 site. In contrast, binding of the classical inhibitors β-CFT and cocaine yielded D79-Y156 distances greater than the 3.5 Å maximum for hydrogen bonding (≈5.5 Å and ≈7.5 Å, respectively), signifying an open vestibular gate in each case. Binding of the atypical inhibitor benztropine, however, resulted in a preserved D79-Y156 hydrogen bond (*i.e.* an interatomic distance less than 3.5 Å), suggesting that—unlike cocaine—binding of benztropine at the S1 site does not prevent closure of the gate.

In an effort to expand upon this finding, we measured the terminal D79-Y156 distance for each of the modeled DAT inhibitors when bound at either the S1 or the S2 site (Figs. 3 and 4, distance values are indicated in yellow at the bottom of each panel). Modafinil docked at the S1 and S2 sites yielded respective D79-Y156 distances of 2.29 Å and 2.25 Å (Fig. 3A–B), suggesting a preserved hydrogen bond and a closed extracellular gating network. Similarly, the atypical inhibitor bupropion gave respective interatomic distances of 2.34 Å and 2.37 Å when docked at the S1 and S2 sites (Fig. 3C–D). In accordance with the findings of Beuming *et al.* (2008), docking of β-CFT at the S1 site resulted in a D79-Y156 distance of 4.85 Å, indicative of an open extracellular gate (Fig. 5A). Interestingly, at the S2 site, extension of CFT's 3β-fluorophenyl moiety downward into the S1 site permitted an aromatic stacking interaction with Y156, pushing the tyrosine ring aside and expanding the D79-Y156 distance to 4.94 Å (Fig. 5B). In addition, the classical inhibitor *d*-methylphenidate also disrupted the D79-Y156 hydrogen bond, yielding S1- and S2-bound distances of 4.12 Å and 3.57 Å, respectively (Fig. 5C–D). This suggests that cocaine-like phenyltropane inhibitors and methylphenidate are capable of inducing an open-to-out transporter conformation upon binding at *either* the S2 or S1 site.

## Discussion

The stimulant and nootropic compound modafinil was initially assumed not to possess a dopaminergic mechanism of action, due to its structural dissimilarity to other DAT ligands and its relatively low micromolar-level affinity for the DAT [71]. However, recent broad-spectrum receptor screening assays have identified the DAT as the only protein target displaying significant (<10 µM) affinity for modafinil (although Madras *et al.* (2006) showed that modafinil also inhibits noradrenaline uptake by the NET, albeit with an IC_50_ value of ≈36 µM) [53]. This is consistent with our finding that modafinil inhibits [^3^H]CFT binding to human DAT with relatively low affinity (*K*_i_ = 2.1 µM). Despite its modest affinity, recent findings that modafinil occupies brain DATs in humans at clinically-relevant doses—and, like any DAT inhibitor, causes an increase in extraneuronal dopamine—have prompted some to proclaim that modafinil may have significant abuse liability, akin to that of traditional cocaine-like DAT inhibitors (*e.g.* [72]). In addition, while certain behavioral studies in animals have shown that modafinil is not self-administered via the IV route and does not induce place preference [73], [74], others have found that high doses of modafinil fully substitute for cocaine in drug discrimination tests [75], [76] and that modafinil occasions conditioned place preference and cocaine-like locomotor sensitization in mice [77].

Clinical and preclinical studies, however, suggest that modafinil neither elicits stimulant-like subjective effects nor encourages self-administration in frequent cocaine users [16], unlike the classical dopamine uptake inhibitor methylphenidate [78]. And while it is widely accepted that interaction with the DAT underlies cocaine's strong addictive potential, extensive research has shown that a number of atypical DAT inhibitors—such as benztropine, GBR12909 and bupropion—have limited reinforcing effects in humans [19], [79], [80], despite fully substituting for cocaine in animal drug discrimination protocols (*e.g.* [76], [81]). Moreover, numerous animal studies have shown that exceptionally potent and selective DAT inhibitors derived from benztropine or GBR12909 incompletely substitute for cocaine in drug discrimination tests and also decrease cocaine self-administration [82]–[85]. As benztropine is also a potent antagonist at muscarinic M_1_ and histamine H_1_ receptors, some have argued that activity at these targets (as opposed to the DAT itself) underlies benztropine's low addictive liability. However, antihistaminergic and antimuscarinic compounds do not attenuate the reinforcing effects of cocaine [86], [87]. Additionally, benztropine analogues with lower affinity for the M_1_ muscarinic receptor than benztropine itself do not exhibit cocaine-like effects [87], making it unlikely that these non-DAT side effects are responsible for the behavioral profile of benztropine and its derivatives. It has also been argued that a slow onset of action (compared to cocaine) is responsible for the non-classical behavioral effects of various benztropine-derived atypical DAT ligands [88], [89]. However, a recent study by Li *et al.* (2011) found that a number of *N*-substituted benztropine analogues possessing rapid onset rates did not induce cocaine-like place preference, suggesting that a slow onset rate is not required for atypical-like behavioral effects [20].

Hence, it appears that addictiveness is not a property shared by all DAT-inhibiting compounds, but instead may be contingent upon a specific sort of molecular interaction with the DAT protein. In this study, we compared the nature of modafinil's molecular interaction with the dopamine transporter to that of characterized cocaine-like and atypical uptake inhibitors, employing a combination of biochemical and computational techniques. There is ample evidence that different classes of DAT inhibitors preferentially bind to (or induce upon binding) distinct transporter conformational states. Experimentally, this idea is supported by the finding that cocaine and benztropine differentially affect the vulnerability of extracellular-facing DAT cysteine residues towards reaction with impermeant sulfhydryl reducing reagents, indicating that these inhibitors stabilize different conformations [40]. In addition, binding of cocaine-like compounds has been shown to protect DAT transmembrane arginine residues from covalent reaction with phenylglyoxal, whereas benztropine-like compounds failed to affect phenylglyoxal reactivity, further hinting at specific conformational effects that vary depending upon the structure of the bound inhibitor [90]. In prior site-directed mutagenesis studies, we identified two DAT mutants (W84L and D313N) that bias the conformational equilibrium of the transporter towards the open-to-out (outward-facing) state [43]. By impeding the transition from open-to-out to occluded and inward-facing conformations, the W84L and D313N mutants enhance the binding affinity of cocaine-like DAT ligands, which bind to and stabilize the outward-facing state. However, the mutations display either unchanged or decreased affinity for atypical inhibitors—as well as DAT substrates (such as dextroamphetamine) and certain bivalent substrate-like ligands (see [47])—allowing them to be used as tools to determine whether or not a particular ligand possesses a cocaine-like mechanism of action. In a previous structure-activity relationship (SAR) investigation of a variety of structurally unique DAT inhibitors, we used these two transporter mutants to show that the presence of a diphenylmethoxy moiety was sufficient (but not necessary) to engender a given DAT inhibitor molecule with an atypical binding profile [42]. This particular functional group is a structural feature common to benztropine, GBR12909 and their respective 3α-diarylmethoxytropane and 1,4-dialkylpiperazine derivatives investigated as therapeutics for cocaine addiction [19]. The fact that modafinil possesses a similar diphenylmethyl structural moiety—albeit with a sulfinyl functionality in place of the diphenylmethoxy ether oxygen atom—was a motivation for investigating its potential conformation-specific interaction with the DAT.

The data obtained with our outward-biasing DAT mutants are consistent with the idea that modafinil exhibits an interaction mode akin to that of the diphenylmethoxy-based inhibitors benztropine and GBR12909, but different than that of cocaine and methylphenidate. That is, like other atypical DAT inhibitors, modafinil preferentially interacts with a “closed-to-out” transporter conformation. This conclusion is further supported by the binding assays we performed under conformation-biasing ionic conditions, as well as our computational modeling data. Amongst the DAT inhibitors tested, the binding affinity of modafinil was the least impacted by replacement of extracellular sodium with the inert cation NMDG, a treatment known to shift the dynamic equilibrium of the transporter from a predominately open-to-out state to a more inward-facing one. Binding of the benztropine analogue JHW007, a potent DAT inhibitor that elicits neither self-administration nor place preference in behavioral reinforcement tests, has also been found to be largely insensitive to extracellular sodium levels [80]. Under these sodium-depleted conditions, “rescue” of the outward-facing transporter state by addition of 10 µM Zn^2+^—which interacts with the DAT above the vestibular S2 site and promotes conformational reorientation from inward- to outward-facing states—dramatically increased the binding of cocaine, β-CFT and methylphenidate, but had no effect on binding of modafinil or the other atypicals (benztropine, bupropion and GBR12909).

In order to provide a structural context for the binding and mutagenesis results, we also performed computational studies of inhibitor interaction with a DAT molecular model. Docking models of β-CFT and dexmethylphenidate demonstrated that these inhibitors promote an outward-facing conformation by breaking a critical D79-Y156 hydrogen bond. By breaking this interaction, cocaine-like inhibitors appear to impede closure of the extracellular gating network and therefore prevent the transporter from transitioning from the open-to-out state to the occluded state. By contrast, docking models of the atypical inhibitors (*R*)-modafinil and (*S*)-bupropion revealed a preserved D79-Y156 hydrogen bond, suggesting that binding of either of these inhibitors does not prevent the DAT from transitioning to a closed-to-out occluded conformation. It is important to note that the respective effects of cocaine-like or atypical inhibitors on the D79-Y156 interaction were maintained when inhibitors were docked in either the central S1 substrate-binding site or the putative vestibular S2 site. The exact binding location of uptake inhibitors in NSS proteins has been intensely debated, particularly following the discovery of tricyclic binding at the S2 site in the bacterial NSS family member LeuT. Our docking models, however, suggest that cocaine-like and atypical inhibitors can exert differential conformational effects in the transporter protein upon binding at either site. Interestingly, the D79-Y156 hydrogen bond is also preserved in models of DAT substrate binding [31], [47]. This raises the possibility that, despite not being translocated across the membrane, atypical inhibitors like modafinil interact with the DAT in substrate-like manner. It has been recently proposed that stabilization of an occluded or inward-facing conformational state, similar to that induced (transiently) during substrate translocation, underlies the ‘cocaine-antagonist’ properties of benztropine and other atypical inhibitors [84]. The rationale being that having a significant percentage of DATs stabilized in a substrate-like closed conformation will prevent cocaine from interacting with the transporter. This idea is in fact consistent with the preclinical literature, which suggests that substrates (such as dextroamphetamine) and atypical DAT inhibitors (such as modafinil and the benztropines) are more effective as treatments for cocaine addiction than methylphenidate, which preferentially interacts with the same transporter conformation as cocaine [91].

## Supporting Information

Figure S1 **Representative clusters of docking poses (“metaposes”) showing potential ligand binding geometries.** Metapose diagrams are shown for the ligands (*R*)-modafinil and β-CFT docked in the S1 site.
(PDF)
