# Correction: The Atypical Stimulant and Nootropic Modafinil Interacts with the Dopamine Transporter in a Different Manner than Classical Cocaine-Like Inhibitors

**DOI:** 10.1371/annotation/ba711a7c-13fb-4d18-89a8-28dcc68fcd04

**Published:** 2012-01-23

**Authors:** Kyle C. Schmitt, Maarten E. A. Reith

There is an error in the funding statement. The correct funding statement is, "Project supported by United States National Institutes of Health (NIH) Grants DA013261 DA019676 and MH083840. The funders had no role in study design, data collection and analysis, decision to publish, or preparation of the manuscript." 

