# Supplementary material for: The Atypical Stimulant and Nootropic Modafinil Interacts with the Dopamine Transporter in a Different Manner than Classical Cocaine-Like Inhibitors
Source: PLoS One. 2011 Oct 17;6(10):e25790. doi: 10.1371/journal.pone.0025790 (PMC3197159; doi:10.1371/journal.pone.0025790)
Supplement: Figure S1 — Representative clusters of docking poses (“metaposes”) showing potential ligand binding geometries. Metapose diagrams are shown for the ligands (R)-modafinil and β-CFT docked in the S1 site. (PDF) [file pone.0025790.s001.pdf]

Supporting Information – The Atypical Stimulant and Nootropic Modafinil Interacts with the Dopamine Transporter in a Different Manner than Classical Cocaine-like Inhibitors

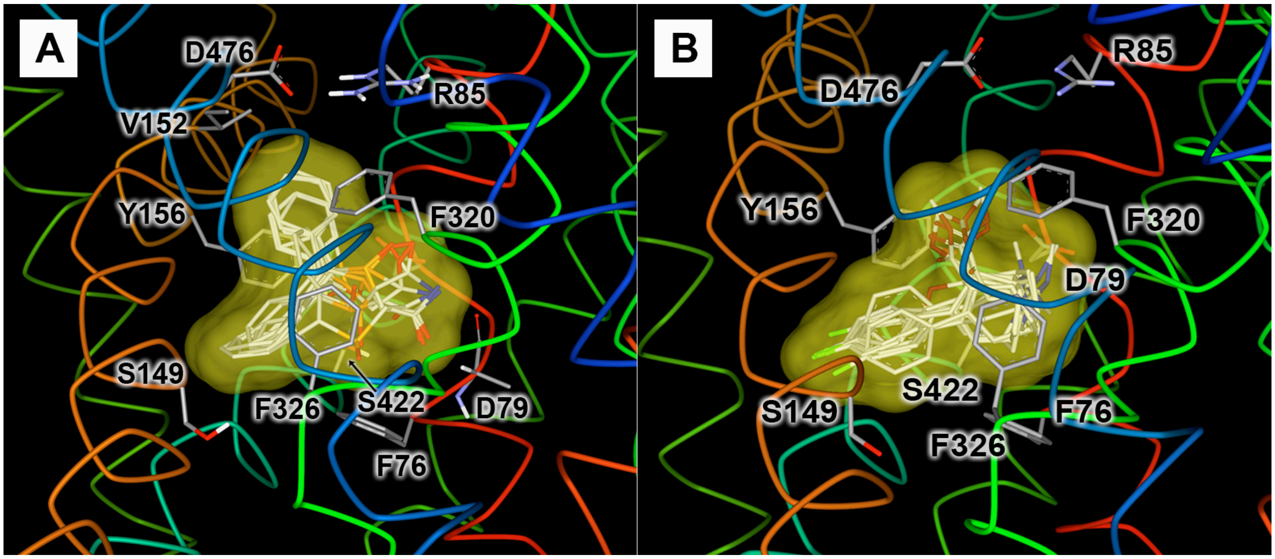

Figure S1. Representative clusters of docking poses ("metaposes") showing potential ligand binding geometries. Metapose diagrams consist of a number of top-scoring ligand orientations from the most highly populated cluster of docking poses shown in the context of a single DAT protein conformation. For each ligand docked at the DAT in either the S1 or S2 substrate-binding site, one representative pose from the cluster was chosen for further protein/ligand complex optimization by sequential rounds of energy minimization. Shown are metapose clusters for the ligands (*R*)-modafinil (**A**) and β-CFT (**B**) docked at the S1 site (corresponding final protein/ligand docking models are shown in Figs. 3A and 5A, respectively). Ligands are rendered as sticks with white carbon atoms and side-chain atoms of selected DAT residues (labeled) are rendered as sticks with gray carbons. Each discrete ligand pose is rendered with a translucent yellow molecular surface (1.4 Å probe radius); hence, the displayed volumes represent the geometric union of all pose surfaces.
